# Neuromorphic Vision Based Contact-Level Classification in Robotic Grasping Applications

**DOI:** 10.3390/s20174724

**Published:** 2020-08-21

**Authors:** Xiaoqian Huang, Rajkumar Muthusamy, Eman Hassan, Zhenwei Niu, Lakmal Seneviratne, Dongming Gan, Yahya Zweiri

**Affiliations:** 1 Khalifa University Center for Autonomous Robotic Systems (KUCARS), Khalifa University of Science and Technology, Abu Dhabi 127788, UAE; rajkumar.muthusamy@ku.ac.ae (R.M.); 100050183@ku.ac.ae (E.H.); 100052634@ku.ac.ae (Z.N.); lakmal.seneviratne@ku.ac.ae (L.S.); Y.Zweiri@kingston.ac.uk (Y.Z.); 2 School of Engineering Technology, Purdue University, West Lafayette, IN 47907, USA; dgan@purdue.edu; 3 Faculty of Science, Engineering and Computing, Kingston University, London SW15 3DW, UK

**Keywords:** robotics sorting, contact-level classification, neuromorphic vision, haptics, machine learning, dynamic vision sensor

## Abstract

In recent years, robotic sorting is widely used in the industry, which is driven by necessity and opportunity. In this paper, a novel neuromorphic vision-based tactile sensing approach for robotic sorting application is proposed. This approach has low latency and low power consumption when compared to conventional vision-based tactile sensing techniques. Two Machine Learning (ML) methods, namely, Support Vector Machine (SVM) and Dynamic Time Warping-K Nearest Neighbor (DTW-KNN), are developed to classify material hardness, object size, and grasping force. An Event-Based Object Grasping (EBOG) experimental setup is developed to acquire datasets, where 243 experiments are produced to train the proposed classifiers. Based on predictions of the classifiers, objects can be automatically sorted. If the prediction accuracy is below a certain threshold, the gripper re-adjusts and re-grasps until reaching a proper grasp. The proposed ML method achieves good prediction accuracy, which shows the effectiveness and the applicability of the proposed approach. The experimental results show that the developed SVM model outperforms the DTW-KNN model in term of accuracy and efficiency for real time contact-level classification.

## 1. Introduction

Robotic sorting is a key part for most industrial production lines. Their applications vary from sorting and organizing products in warehouses, automotive assembling in manufacturing plants, and cleaning up debris in disaster zones. Utilizing robots for sorting can effectively reduce labor intensity, space saving, and easy to re-deploy for various applications. Besides, it gives the industry the advantage of reducing the production time while increasing the throughputs. In the 4th industrial revolution, the demand on robots for doing multiple tasks is highly increased. The bulk of these tasks require the robots to be proficient in gripping objects with different shapes, weights, and textures. However, the majority of present techniques are used to train robots to perform tasks that are suitable for a structured environment. Such tasks are prone to high error and are tremendously difficult to be fully automated, especially for unstructured environments [1]. In order to deal with different situations, such as unstructured environment, robotic sorting based on vision has huge advantages on dealing with changes of working environment [2].

Capitalizing on that, several approaches have been suggested over the past decades to improve grasping behavior for robot sorting applications [1,3]. A range of applications utilizing the grasping techniques has been devised, ranging from micro scales to macro scales [1]. For instance, work in [4] exploited robot grasping ability in an automatic system for sorting garbage. It achieved a sorting goal that is based on recognizing target shapes and position utilizing the Region Proposal Generation (RPN) and the VGG-16 model. Work in [5] designed a self-adopting claw for sorting apples, which can adjust grasping force by regulating the angular displacements of hinges according to sizes of apples. Recently, soft-gripper is employed in industry for sorting applications [6,7]. Soft gripping has the flexibility to adapt for different object shapes and hardnesses when compared to the hard gripping method, which makes it preferable for other purposes that require a sensitive grasping. Besides, utilizing soft gripper is a useful way to reach a better grasping. Nevertheless, soft gripper requires a complex structural design, dynamic modelling, and gripper control. Thus, sensing capability is important to soft robotic grippers to precisely handle or classify objects. In our work, we use soft finger and contact level decision making. In particular, we focus on how vision based tactile sensing facilitates contact level decisions for soft gripping robots. In addition, through the deformation of silicon wafer, objects’ characteristics, such as size and shape, can be observed by the camera that is crucial for improving system performance.

For precise grasping, different types of sensors were developed for sensing tactile signal, such as capacitive sensors [8] and piezoresistive sensors [9]. Despite the extensive research that has used various types of tactile sensors for grasping action, they still lack the proper spatial and temporal resolution. Moreover, they are also limited by large sizes, high hysteresis, and interference with other electronics. Progression in image processing techniques and optical technology over the past decades has a significant impact on improving robots grasping for sorting purposes. A vision-based sorting approach is proposed in [10] to group similar parts while using Bayesian estimation. Recently, interesting applications, such as garbage sorting [4], transparent Plastic Granulate sorting [11], and material sorting [12], are proposed utilizing different vision-based techniques. The advantage of vision-based tactile sensor resides in its ability to provide a high resolution. However, processing images is often involved with a lot of redundant pixels, which adds further computation and memory requirement. Therefore, a neuromorphic event-based camera (Dynamic and Active-Pixel Vision Sensor (DAVIS) 240C) [13] is adopted for this research work. Due to its ability to provide dense temporal information about the changes in scene, an accurate and fast detection becomes achievable in the dynamic environment. Therefore, the unique property of DAVIS becomes indispensable to improve the performance for grasping in sorting applications. To that end, few works employed DAVIS for tackling grasping behaviors, such as dynamic force estimation [14] and incipient slippages detection and suppression [15,16]. In this work, we explore and study how the event based tactile sensor with occluded skin can be effective in contact-level classification, especially in robotic sorting applications.

The main advantages of event-based camera are of local gain control, sparse output, low latency and non-motion blur. In addition, it has a high dynamic range (140 dB) with low-power consumption (10 mW) as compared to the traditional camera. DAVIS is used to measure the pre-pixel brightness changes in the scene asynchronously. The resulting stream of events have a microsecond resolution, encode time, address, and the sign of brightness changes, called polarity [13]. Therefore, when compared with traditional visual tactile sensor, utilizing events-based grasping has the advantages of higher sampling rate and faster response. Building on that, the prior information of objects can be obtained with a low latency that can effectively improve grasping performance. This work aims to classify object size, material hardness, and grasping force based on sequential events information to develop grasping prior knowledge. The grasping is only considered to be successful when classifications reach a high accuracy. Otherwise, the gripper will re-adjust its position and orientation to re-grasp the object properly. Moreover, prior knowledge can be helpful for other grasping application by estimating the initial force for reliable grasping by classifying object size, material hardness and contact force based on events. Accordingly, a stable lifting would be achieved with less and even no slippage, as well as without any damage. Besides, the guidance and rules of force control during grasping and manipulating would also be provided.

However, the deformation of silicon wafer under pressure is highly non-linear. It depends on the range of the applied force beside the shape and the hardness of the contacted object. Additionally, other factors can affect this relationship, such as the temperature of the membrane and the sensor light intensity. Accordingly, non-linear relationships among triggered events and grasping force, objects size, and material hardness exits. The correlation between accumulated events and the image of the contact force over a pre-defined time interval is visualized in Section 4. Robust ML approaches are adopted to capture the non-linear relationship over time in this work to obtain prior knowledge of the object characteristics based on DAVIS triggered events. The existing ML methods for sequence classification are categorized as feature based, sequence distance based, and model based classifications [17]. Amongst them, sequence distance based methods are widely used and adopted for time series classification. Particularly, SVM and KNN-DTW are superior for classification precision when compared to other methods. SVM is a powerful method for building a classifier that aims to create a decision boundaries between classes, so it enables the prediction of labels from one or more feature vectors [18]. The SVM methodology has been successfully applied in many applications such as genomics, financial data analysis, signal processing, and time series classifications, due to its robustness for estimating predictive models from noisy, sparse, and high-dimensional data [19]. Moreover, the similarity between time sequences is measured in the time-series classification problem. The most popular methods calculate Euclidean distance to estimate the similarity, but they cannot find the best alignment between time series. Dynamic Time Warping is well known for measuring the similarity between two series in timing. It has been widely used in many fields, such as data mining [20,21], gesture recognition [22], robotics [23], speech processing [24,25], and medicine [26]. Besides, K-Nearest Neighbor is an unsupervised method of clustering, so it can be used as a density-based classifier. K Nearest Neighbor has been successfully used in many applications, including handwritten digit recognition [27] and gene expression classification [28]. Building on that, KNN is integrated with DTW for object’s prior knowledge classification in this work.

To improve the performance of robotic sorting in industry, we propose a novel neuromorphic vision based approach to overcome limitations of conventional cameras. Besides, a Machine Learning approach utilizing SVM and DTW-KNN is developed for contact-level classification, in order to acquire the prior knowledge of objects based on EBOG datasets created. Realizing the new direction of parasitism theory [29] for evolving technology to explain the complex relationships between variables in the systems, we can consider the robot robotic sorting as the host system, and event-based robotic grasping and contact-level classification are the parasitic systems. In addition, the results for both methods are compared for further real-time implementations. The contributions of this paper are summarized, as following:

- a novel approach utilizing the developed neuromorphic vision based tactile sensor is developed for contact level classification
- machine learning approaches utilizing SVM and DTW-KNN are developed to classify material hardness, object size and grasping force. The classification accuracy indicates whether the object is sorted successfully, and also has a paramount effect in helping gripper to re-adjust and re-grasp to ensure a successful grasping and sorting;
- after conducting 243 experiments, an Event-Based Object Grasping (EBOG) dataset are generated. To date, this is the first events dataset generated to analyze the grasping behavior in robotic applications; and,
- scenarios of neuromorphic vision based robotics sorting in structured and unstructured environment are presented.

In the following sections, we introduce the design of neuromorphic event-based tactile sensor and EBOG dataset created. ML approaches including SVM and DTW-KNN are addressed in Section 3. The results of classification based on different approaches are presented and discussed in Section 4. In addition, scenarios of robotic grasping and sorting applications based on events camera is illustrated in Section 5. Conclusions of this work and the future work are discussed in Section 6.

## 2. Event-Based Object Grasping Dataset

**Figure 1:**
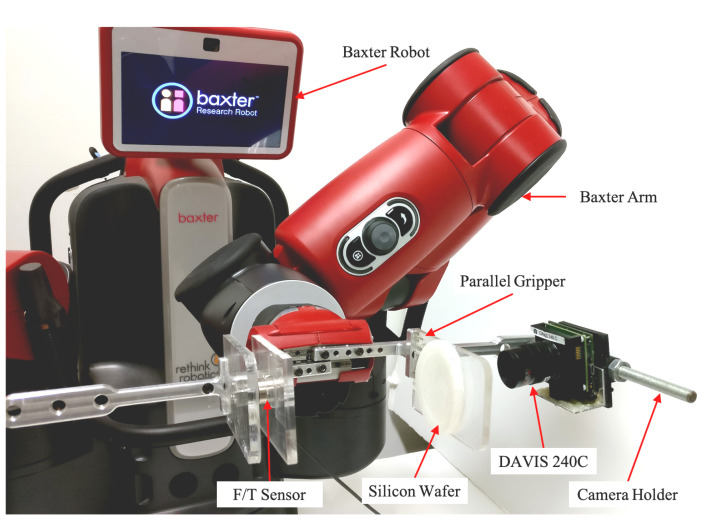
Experiment setup with neuromorphic event-based tactile sensor.

To build and train machine learning classifiers, the EBOG dataset is generated through conducting 243 set of experiments utilizing event-based camera for robotic grasping, holding, and releasing. The neuromorphic vision-based tactile sensor is employed and positioned on the right side of Baxter’s gripper, as illustrated in Figure 1. The Baxter robot has two arms that each arm consists of seven joints and each joint has two degrees of freedom (DOF). A parallel gripper system is designed for Baxter robot, which includes the metallic and the acrylic part. The metallic gripper is designed with an adjustable camera holder and mounted on Baxter’s arm, which can enable a stable grasping and eliminate the vibration due to the Baxter gripper elasticity. A camera holder is essential to help DAVIS detect the interested area by adjusting its position and orientation. Besides, the transparent acrylic material is attached to the electrical gripper, which helps in grasping the object. Its transparency allows for DAVIS to observe the changes in the grasped object without occlusion. In addition, the ATI F/T sensor (Nano17) is attached on the left gripper to measure the contact force at each time interval, which is used as a ground truth to trace object grasping, holding and releasing phases. In addition, the soft material-silicon wafer is attached on the inner side of the right gripper to bring a certain flexibility to gripper. DAVIS is a dynamic active-pixel vision sensor, which captures per-pixel illumination changes as events for moving object asynchronously. The stream of events encodes time *t*, position (x,y), and polarity *p*. To enhance the gripper’s ability to grasp objects with different sizes and shapes to a certain degree, a semi-transparent silicon wafer is attached on the inner side of the right gripper, as mentioned in Section 1. Moreover, it ensures DAVIS camera’s ability to detect the tiny changes at the contact surface during silicon deformation.

**Figure 2:**
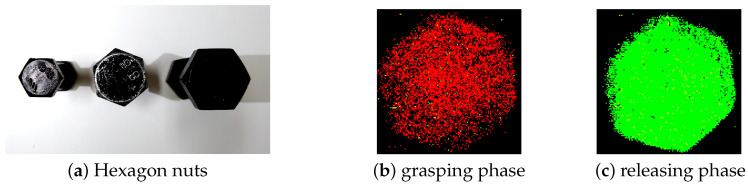
(**a**) Hexagon nuts with different sizes and images of events in grasping phase and releasing phase. (**b**) red spots represents negative-polarity events which are dominant in grasping phase.(**c**) green spots represents positive-polarity events, which are dominant in releasing phase.

In this work, nuts are used as the sorting target, which are the basic and essential elements for industrial machines and products [30]. Picking up, recognizing, and sorting various shapes of nuts are not a difficult but tedious task for human, but they are actually pretty difficult tasks for machines and robots. Though nuts come in thousands of shapes and sizes, hexagon nuts are the most common ones that used for industrial as well as commercial use. Therefore, this work aims to sort hexagon nuts, as shown in Figure 2a, according to their sizes. In this experiment, nuts with small (11 mm), medium (13 mm), and large (17 mm) size are used for grasping and sorting. The grasping force sets as 10%, 50%, and 100% of gripper’s maximum grasping force. Moreover, the hardness of silicon wafer also varies in the range of small, medium, and large degrees with thicknesses of 4 cm, 7 cm, and 10 cm, respectively. For each setting condition, 9 experiments were conducted for the certain object size, grasping force, and silicon hardness. Individual experiment includes three phases: grasping phase, holding phase, and releasing phase. Therefore, total 243 (9×3×3×3) sets of experiments data are obtained under different conditions.

In grasping phase, the gripper closes in order to cage the object until the pre-defined gripping force is reached. Simultaneously, the negative events, as shown in Figure 2b increase due to the reduction in the light intensity. Subsequently, the object is held with the same grasping force for some duration. In the last phase, the gripper moves back to the original position to release the object. Due to losing contact between the object and the silicon wafer during gripper’s opening, more events with a positive polarity are triggered, as shown in Figure 2c.

**Figure 3:**
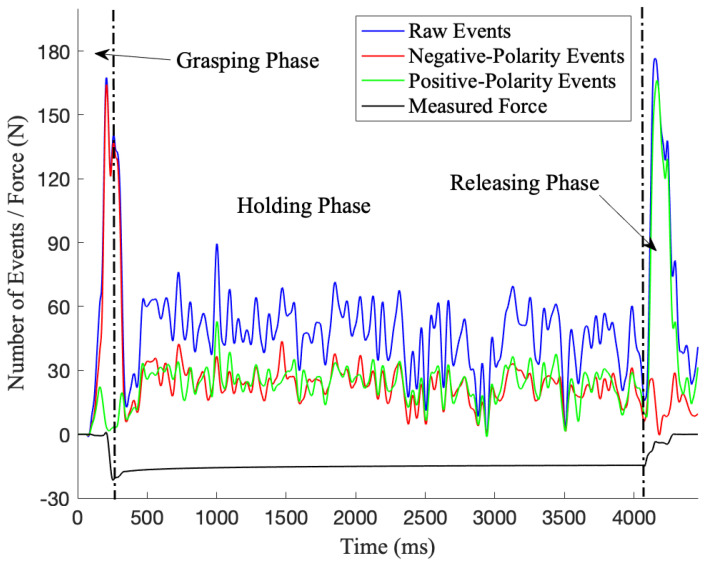
Events data of a single experiment which consists of grasping, holding and releasing phases. Raw events represents the total amount of events in 1 ms interval, including both positive and negative events.

Recognizing the inherent properties of DAVIS, which enables detecting events at the microsecond level. Therefore, any small source of noise, such as gripper vibration, sensor temperature, and lighting environment, would lead to a large effect on signal-to-noise ratio (SNR). Therefore, events are framed over 1 ms to alleviate the noise impact. In this work, 243 sequences of raw, positive, and negative events data are collected to the EBOG for all three phases. Figure 3 illustrates the number of events for a single experiment over time. As depicted in Figure 3, the absolute contact force measured by F/T sensor changes significantly in grasping and releasing phase, which is used to define and trace these three phases. It is apparent that negative events and positive events are dominant in grasping and releasing phases, respectively. The first peak in negative events represents the first touch between object and silicon wafer, which indicates the increase in the contact force at the contact level. Similarly, the highest peak of positive events indicates that the gripper is losing contact with the object in releasing phase. Moreover, it can be observed that the raw events, positive and negative events fluctuate within some range in the holding phase, due to the gripper’s vibration and noise in the vicinity. Hence, the triggered events in the grasping phase represents the most valuable information for grasping and sorting.

Therefore, events of the grasping phase are the main focus for robotic grasping and sorting in this work. From Figure 3, it is apparent that the amount of negative-polarity events shows the most dramatic change, carrying the most significant and meaningful information. Thus, sequences of negative events of the grasping phase is applied as input for prior knowledge classification. Building on that, three variables, which are the grasping force, the size of object, and the hardness of silicon wafer are the main targets for classification. In addition, the objects prior knowledge is required to be known in the early stage of grasping. Classification accuracy is not only results for sorting objects, it is also used as a metric for gripper’s decision on re-adjust and re-grasp to ensure a successful grasping and sorting.

## 3. Machine Learning Approach

For acquiring the contact-level information in the grasping phase, two machine learning approaches: SVM and DTW-KNN are investigated in this work. The amount of negative events, which represents the most significant information and collected during grasping phase, are used as the classifier input. 243 sequences of negative events data are obtained and prepared through experiments. 189 (78%) and 54 (22%) sets are used for training and testing classifiers, respectively. Limited by the small number of time series utilized for classification, general deep learning methods would be easily prone to over-fitting. Therefore, machine learning approaches, a well known method that is capable to handle the small dataset with reasonable accuracy, are used to tackle this problem. SVM is usually a successful approach for time series classification and prediction, on account of its ability to handle the non-linear relationship between parasite-host systems. Besides, DTW-KNN is also implemented in order to handle the time characteristics of time series. Classifiers of SVM and DTW-KNN will be compared according to classification results, then the one with better results will be nominated for the future real-time sorting application.

### 3.1. Support Vector Machine

SVM is a supervised learning method, which aims to clearly classify diverse classes by optimal hyperplanes which are also called as decision surfaces. In this work, SVM with Gaussian kernel is used to classify non-linear events data. The expression of Gaussian kernel is

(1) $$k\left(x,x^{\prime }\right)=e^{\left(-||x-x^{\prime }|{|}^{2}/2\sigma^{2}\right)}$$

where *x* and $x^{\prime }$ are two feature vectors, and σ is related to the fitting degree of SVM model. Hyperplanes of SVM classifier can be generally represented as:

(2) $$f=\sum_{i=1}^{n}w_{i}\cdot y_{i}\cdot k\left(x_{i},x\right)\pm b$$

where $w\in R^{n}$, b∈R, *n*, $x_{i}$, and $y_{i}\in \left\{-1,+1\right\}$ denote weight, bias, size of training data, the support vector, and the corresponding output, respectively. The region bounded by hyperplanes is called the separation “margin”, which is given by

(3) $$\rho =\frac{2}{\sqrt{w^{T}w}}$$

The goal is to find the optimal values of *w* and *b* that can maximise the margin ρ.

### 3.2. KNN-DTW

**Figure 4:**
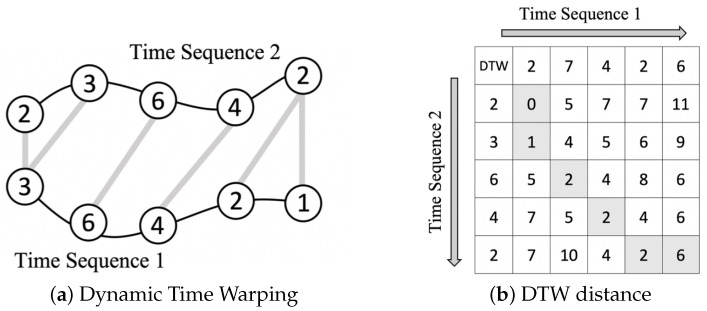
The principal of DTW. (**a**) The best alignment of two time sequences by DTW. (**b**) The distance matrix of finding the DTW optimal path of alignment.

KNN is a density-based unsupervised classifier, which classifies unlabeled data according to the majority vote of *k* nearest labeled data. Before testing, the optimal value of *k* is selected through conducting several trials with different values. Euclidean distance is generally used to present the nearest distance in KNN. However, it ignores the dynamic characteristics of the time in time-series. Therefore, DTW is applied in order to calculate the similarity between time sequences, which may vary in speed. The principal of DTW is illustrated in Figure 4 and the best alignment shown in Figure 4a can be obtained by calculating the distance matrix, as demonstrated in Figure 4b.

**Figure 5:**
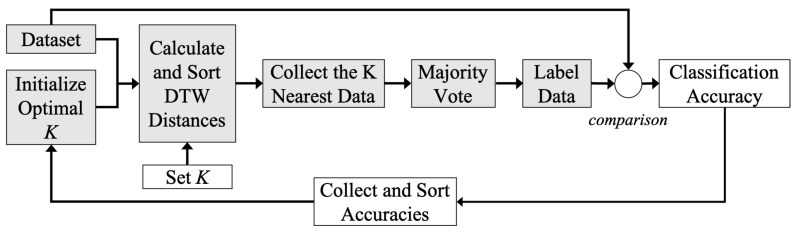
The working flow chart of DTW-KNN classifier which includes two processes: *k* selection described in both black and white blocks, and DTW-KNN classification described in black blocks.

The distance of each point can be evaluated by adjacent points:

(4) $$D\left(i,j\right)=D\left(x_{i},y_{j}\right)+\min\left\{\begin{array}{c} D(i-1,j)\\ D(i,j-1)\\ D(i-1,j-1) \end{array}\right.$$

where D(i,j) is distance between two time sequences with the best alignment and D(i−1,j), D(i,j−1), and D(i−1,j−1) are the neighborhood cells. $x_{i}$ and $y_{j}$ are data in two different time sequences, respectively, and $D(x_{i},y_{j})$ is the distance between $x_{i}$ and $y_{j}$. Figure 5 depicts the processes of *k* selection and DTW-KNN classification.

Blocks in gray belong to both processes of choosing *k* and testing classifier. After loading the dataset, the DWT distance between two time series at a particular point is computed instead of Euclidean distance. Then according to the majority vote from *k* neighbors, the unknown data can be directly labeled. Blocks in white represent additional parts of optimal *k* selection. The classifier is executed several times with different setting values of *k*. By comparing the voted labels to true labels, the classification accuracy under conditions of different *k* are listed. Therefore, the optimal *k* is selected as the one with the highest classification accuracy.

## 4. Contact-Level Classification

**Figure 6:**
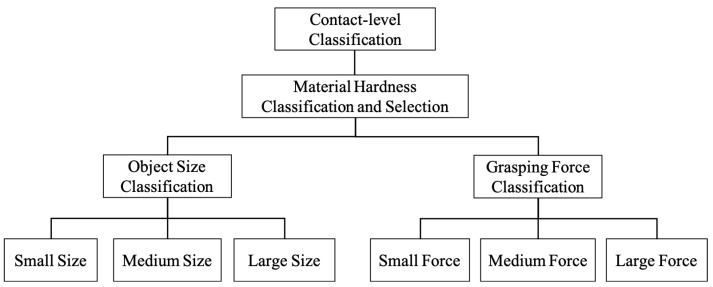
The tree diagram of contact-level classification.

As mentioned in Section 1, the contact-level classification is an useful tool for acquiring prior knowledge of targets in industrial applications such as sorting objects. The tree diagram contact-level classification process is illustrated in Figure 6, the material hardness is classified and selected first due to its nonlinear relationships with other variables and events. Subsequently, object size and grasping force are independently classified under the specific material hardness selected according to events. In this section, the classification processing by SVM and DTW-KNN approaches and the results are discussed.

### 4.1. Selection of Silicon Hardness

In this paper, three variables: object size, contact force, and silicon hardness are the main concern for neuromorphic vision-based contact-level classification. However, the silicon wafer shows different degrees of deformation under different object sizes and contact forces. For instance, the amount of events increases when the hardness of silicon wafer decreases. This happens, since the softer material deforms more severely when applying the same force. To reduce the complexity of relationships among events and other variables, the hardness of the silicon wafer is determined first.

Machine learning approaches based on SVM and DTW-KNN are developed in order to classify the hardness of silicon wafers utilizing events captured during the grasping phase and collected to the EBOG dataset. As mentioned in Section 2, the input of classifiers is time sequences of the amount of negative-polarity events, which is accumulated over every 1 ms interval in the grasping phase. The results of hardness classification using SVM and DTW-KNN classifier are displayed in Table 1 and Table 2, respectively. Becasue both precision and recall values should be high as possible, it is difficult to compare the models with low recall and high precision or vice versa. Therefore, ***F1 Score*** is used to measure both ***Precision*** and ***Recall*** simultaneously. ***F1 Score*** is calculated as Harmonic mean of ***Precision*** and ***Recall*** that F1score=2*((precision*recall)/(precision+recall)).

It can be observed that SVM outperforms DTW-KNN in silicon hardness classification from Table 1 and Table 2. Both of the methods provide consistent results in the classification of the softest silicon wafer classification which attains the best performance for all metrics. Additionally, the F1 score for class 0 (10% material hardness) reaches 100% and 82.1% using SVM and DTW-KNN sequentially, which is the highest among all three hardnesses. This is due to the nature of soft material that deforms acutely during grasping. Consequently, more valuable information of negative events can be captured by DAVIS with softer silicon wafer. Therefore, it manifests that the material hardness is one of the crucial elements for contact-level applications utilizing DAVIS, which can directly affect on the amount of information to be detected. Generally, the softer material can provide more details for DAVIS due to more events triggered accordingly. Hence, in this work the experimental data under the condition of the softest silicon wafer with 4 cm thickness is used for classifying the other two variables. Capitalizing on that, the softer material is more suitable for applications that require detailed information and high observing sensitivity, such as grasping slip detection. Nonetheless, the thicker soft material, which can filter out some details, are preferable for applications that demand low noise environment.

### 4.2. Contact-Level Classification

**Figure 7:**
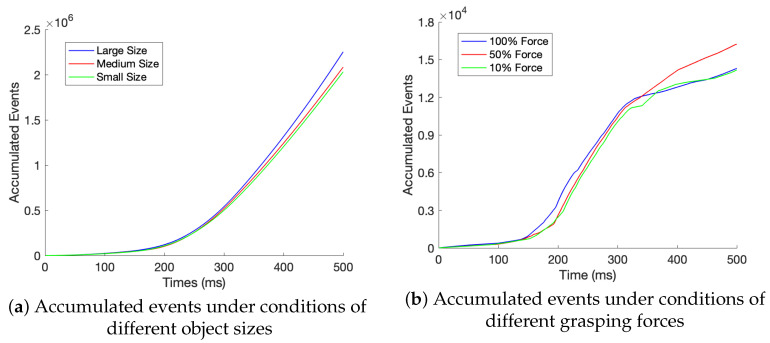
The negative events in the grasping phase are accumulated with time for a certain type of sizes or forces.

For sorting application, the contact-level classification is the core part. The negative-polarity events that are obtained for softest material are used as the input to classify the object size and the grasping force, respectively. Because of the one-to-many relationships between inputs and outputs, single classifiers for each variable are developed, which can provide more detailed information of complex and correlated relationships between each variable and events. As explained in the previous subsection, the object size and grasping force classifications examined under the conditions of 10% silicon hardness. Subsequently, 81 (9×3×3×1) sequences of events data for different grasping force and object size are used. For observing more intuitive relationships between variables, 27 (9×3×1×1) sequences of negative events for individual experiments under the same condition are accumulated and traced, as shown in Figure 7. Figure 7a depicts behaviors of accumulated negative events when grasping objects of small, medium, and large sizes. Similarly, trends of accumulated negative events when grasping objects with 10%, 50%, and 100% of maximum grasping forces are plotted in Figure 7b. A consistent behavior of accumulated events under different object sizes is observed from Figure 7a; however, the behavior of accumulated events for forces depicted in Figure 7b lacks a consonant pattern.Therefore, data preprocessing is required before classification for building and training ML models. When compared with the whole pattern of 500 ms, it is obvious that the accumulated data before 340 ms reveals a similar pattern in Figure 7a. Therefore, the sequences of data in the duration of 0 to 340 ms are used for grasping force classification.

**Figure 8:**
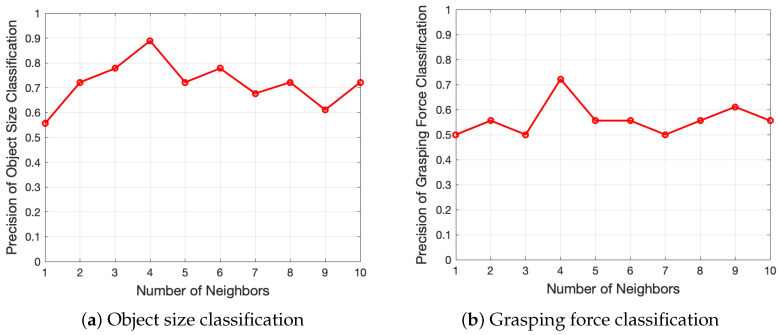
Relationships between KNN classification accuracy of object and grasping force with different values of nearest neighbor data for majority vote.

For size and force classifications, the SVM and DTW-KNN approaches are employed. 63 out of 81 sequences of data are randomly selected for training, and the remaining 18 sequences are used for testing. To obtain compatible results, the same training and testing data of SVM classifiers are utilized as labeled and unlabeled data to build DTW-KNN model. In the DTW-KNN approach, values in range of 1 to 10 are tested in order to initialize *k* value of neighbored data for majority voting. The highest accuracy is reached when 4 neighbored data are included for majority vote, as shown in Figure 8. Therefore, k=4 is selected in the DTW-KNN approach for object size and grasping force classifications.

In performance evaluation, classification accuracy and elapsed time are the metrics used for SVM and DTW-KNN approaches. The accuracy of classifiers that are trained by different approaches are shown in Table 3. Support Vector Machine and Dynamic Time Warping-K Nearest Neighbor obtain the same accuracy (88.9%) for object size classification. However, from Table 4, the metrics, including Precision, Recall, and F1 Score demonstrate that SVM performs better in object size classification. For grasping force classification, the SVM classifier outperforms the DTW-KNN classifier with accuracies of 77.8% and 72.2%, respectively. However, both DTW-KNN and SVM present poor performance of grasping force classification when compared to size classification. Moreover, the soft finger utilizes silicon material as the skin interface; such an interface deforms upon contact and exhibits a highly nonlinear relationship between observed events and variables. Thus, it impacts the object size and grasping force classification accuracy.

Besides, the elapsed time of the prediction model is used in order to assess performances of SVM and DTW. Table 5 shows the elapsed time of DTW-KNN keep high in both cases. However, the elapsed time of SVM is approximately 10 times smaller than the one of DTW-KNN. According to the observed results, the SVM model is more efficient and has the potential to be used in real-time sorting applications.

## 5. Sorting Application Scenario

Integrating event-based contact level classification and neuromorphic vision based tactile sensor, structured and unstructured sorting applications can be targeted. Objects are highly organized in the structured sorting task. Subsequently, robots can easily implement the sorting task according to the presetting information. However, for unstructured sorting, a small change of objects, such as the orientation, will probably affect robotics grasping and sorting result. Thus, robots are required to make a decision on whether re-grasp and re-adjust, in order to ensure an efficient grasping and a successful sorting in an uncertain environment.

These two types of robotic grasping and sorting tasks can be implemented based on the prior knowledge obtained by the contact-level classification according to EBOG dataset. The sorting application consists of three phases: caging, grasping, and re-adjusting, and dropping. In operating platform, there are three objects with small, medium and large size are placed in front of the gripper. Besides, three corresponding boxes are placed on the side of it. The goal of this sorting task is to automatically pick up a nut, perform events-based classification, and drop it into the corresponding box.

**Figure 9:**
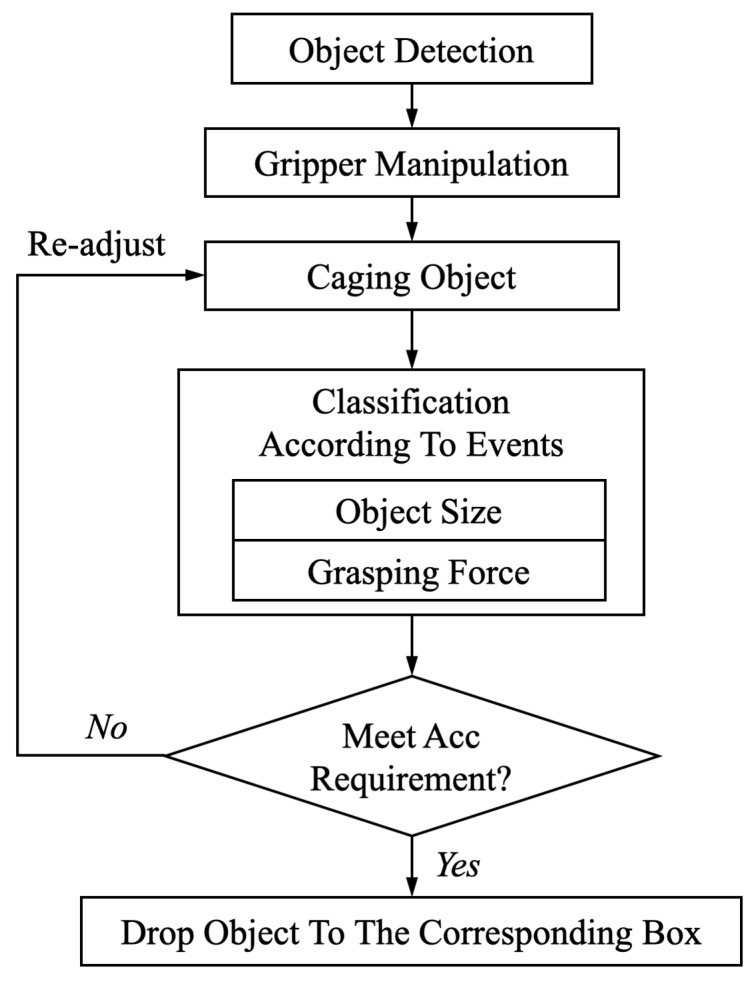
The flow chart of sorting application’s scenario

**Figure 10:**
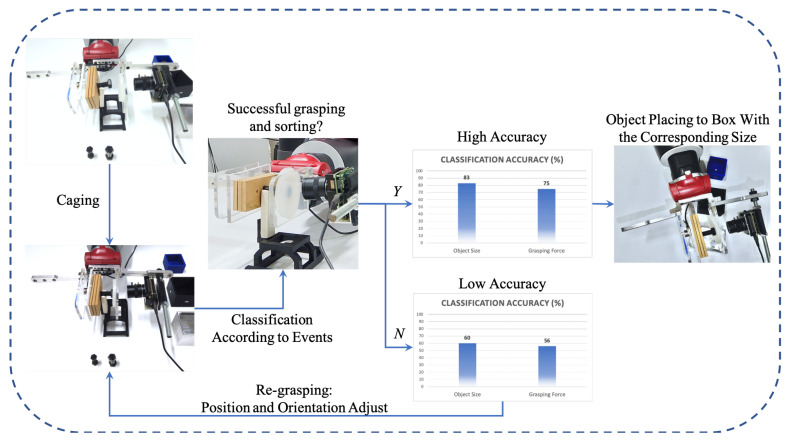
Three phases of sorting application. The large-size nut object is placed on the holding base to be grasped, then object size and grasping force are classified simultaneously during caging. According to classification results, gripper makes decision on re-grasping or placing the object.

Figure 9 describes the flow chart of the sorting scenario. Firstly, the position information of the object will be obtained via object detection techniques. According to the object’s position, gripper will move to a proper place for caging.In this work, the contact-level classification is focused. Accordingly, an ideal and simplified scenario is assumed, as shown in Figure 10, where object detection and gripper manipulation have already been implemented. Subsequently, in caging phase, the object size and grasping force will be classified simultaneously. If the accuracy does not meet the requirement due to the improper grasp such as the misaligned grasp and the incomplete view of object for camera, it will be considered to be an unsuccessful caging. In addition, the wrong classification of object size would directly result in a failure to the sorting task. Accordingly, the grasping will be re-adjusted until the the object is properly gripped and high accuracy of classifications is reached. Subsequently, dropping the object into the corresponding box according to the classified object size.

## 6. Conclusions

In this paper, events camera DAVIS is utilized to develop the neuromorphic vision-based tactile sensor for sorting application. DAVIS has low latency and low power consumption when compared to conventional cameras. In addition, two ML methods, SVM and DTW-KNN, are developed to classify object size, grasping force, and material hardness. A special type of EBOG dataset is generated by conducting 243 experiments for training the classifiers. The material hardness is first selected in this work due to the complex relationship with events. According to the three-level hardness prediction, the softest silicon wafer is used for contact level classification, as it provides the highest classification accuracy (100%). It manifests that severe deformation of softer material results in a high sensitivity of observation. However, for other works that detail information is not necessary or noise is required to be filtered, the thick material with higher hardness will be suitable due to its light deformation. Moreover, soft skin interface deforms upon contact that makes objects size and grasping force classifications challenging. Both SVM and DTW-KNN approaches used to classify object sizes and provide a same accuracy (88.9%), but SVM classifier performs better on metrics of Precision, Recall, and F1 Score. Moreover, the trained SVM model provides a more precision result (77.8%) for grasping force as compared to DTW-KNN. When considering the elapsed time aspect, the SVM method has more potential for real-time applications.

This event-based contact-level classification benefits robotic sorting for its high sampling rate, which enables the robot to respond faster and re-adjust grasping to ensure a successful action. In addition, contact-level classification can be used for initial grasping force estimation and slip detection to improve the grasping performance in robotic sorting. For future work, the accuracy of the object size and grasping force classifications by SVM and DTW-KNN can be improved. Both approaches involve supervised methods, which are limited to classify the same or similar objects, as being used to infer a function from labeled training data by mapping inputs and outputs. Therefore, it is suggested to use unsupervised approach, Spiking Neural Networks (SNNs), to improve classification performance in future work. Additionally, developing a data augmentation technique for time series will help to avoid the over-fitting problem.

## Figures and Tables

**Table 1:** Silicon hardness classification by SVM. Class 0, Class 1 and Class 2: silicon wafer with 10%, 30% and 50% hardness. *Support*: the number of samples in each class. *Acc*: the ratio of samples that are classified correctly out of all the classes. *Precision*: the percentage of each correctly classified label. *Recall*: the ratio of correctly classified samples out of all the positive classes. *F1 Score*: Harmonic mean of *Precision* and *Recall*.

Terms	Precision	Recall	F1 Sore	Support	Acc
Class 0	100%	100%	100%	18	-
Class 1	70%	77.8%	73.7%	18	-
Class 2	75%	66.7%	70.6%	18	-
Ave/Total	81.7%	81.5%	81.4%	54	81.5%

**Table 2:** Silicon hardness classification by DTW-KNN. Class 0, Class 1, and Class 2: silicon wafer with 10%, 30%, and 50% hardness. *Support*: the number of samples in each class. *Acc*: the ratio of samples which are classified correctly out of all the classes. *Precision*: the percentage of each correctly classified label. *Recall*: the ratio of correctly classified samples out of all the positive classes. *F1 Score*: Harmonic mean of *Precision* and *Recall*.

Terms	Precision	Recall	F1 Score	Support	Acc
Class 0	88.9%	76.2%	82.1%	18	-
Class 1	77.8%	73.7%	75.7%	18	-
Class 2	55.6%	71.4%	62.5%	18	-
Ave/Total	74.1%	73.8%	73.4%	54	74.1%

**Table 3:** Comparison of classification accuracy of object size and grasping force in case of softest silicon wafer by SVM and DTW-KNN.

Testing Accuracy	DTW-KNN	SVM
Object Size Classification	88.9%	88.9%
Grasping Force Classification	72.2%	77.8%

**Table 4:** Comparison of object size classification results by DTW-KNN and SVM. Support: the number of samples in each class. Acc: the ratio of samples that are classified correctly out of all the classes. Precision: the percentage of each correctly classified label. Recall: the ratio of correctly classified samples out of all the positive classes. F1 Score: Harmonic mean of Precision and Recall.

Terms	Precision	Recall	F1 Sore	Support	Acc
DTW-KNN	91.1%	91.1%	69.2%	54	88.9 %
SVM	92.6%	91.7%	91.1%	54	88.9%

**Table 5:** Elapsed time of training model and prediction by SVM and DTW-KNN for object size and grasping force classification.

Elapsed Time (s)	Object Size Classification		Grasping Force Classification	
	SVM	DTW-KNN	SVM	DTW-KNN
Training Model	0.039278	0.603076	0.033888	0.335808
of Prediction	0.025083	0.283062	0.011765	0.177691

## Abbreviations

The following abbreviations are used in this manuscript:

DAVIS	Dynamic and Active-Pixel Vision Sensor
ML	Machine Learning
SVM	Support Vector Machine
DTW	Dynamic Time Warp
KNN	K Nearest Neighbor
EBOG	Event-Based Object Grasping
